# The predictive value of anthropometric indices for cardiometabolic risk factors in Chinese children and adolescents: A national multicenter school-based study

**DOI:** 10.1371/journal.pone.0227954

**Published:** 2020-01-21

**Authors:** Yamei Li, Zhiyong Zou, Jiayou Luo, Jun Ma, Yinghua Ma, Jin Jing, Xin Zhang, Chunyan Luo, Hong Wang, Haiping Zhao, Dehong Pan, Peng Jia

**Affiliations:** 1 Department of Maternal and Child Health, Xiangya School of Public Health, Central South University, Changsha, Hunan Province, China; 2 Institute of Child and Adolescent Health, Peking University School of Public Health, National Health Commission Key Laboratory of Reproductive Health, Beijing, China; 3 Department of Maternal and Child Health, School of Public Health, Sun Yat-sen University, Guangzhou, Guangdong Province, China; 4 School of Public Health, Tianjin Medical University, Tianjin, China; 5 Department of School Health, Shanghai Municipal Center for Disease Control and Prevention & Shanghai Institutes of Preventive Medicine, Shanghai, China; 6 School of Public Health and Management, Chongqing Medical University, Chongqing, China; 7 School of Public Health and Management, Ningxia Medical University, Yinchuan, Ningxia Hui Autonomous Region, China; 8 Liaoning Health Supervision Bureau, Shenyang, Liaoning Province, China; 9 Faculty of Geo-information Science and Earth Observation, University of Twente, Enschede, The Netherlands; 10 International Initiative on Spatial Lifecourse Epidemiology (ISLE), Enschede, The Netherlands; Loyola University Chicago, UNITED STATES

## Abstract

**Objectives:**

This study aimed to assess the accuracy of body mass index (BMI) percentile, waist circumference (WC) percentile, waist-height ratio, and waist-hip ratio for identifying cardiometabolic risk factors in Chinese children and adolescents stratified by sex and BMI categories.

**Methods:**

We measured anthropometric indices, fasting plasma glucose, lipid profile and blood pressure for 15698 participants aged 6–17 in a national survey between September and December 2013. The predictive accuracy of anthropometric indices for cardiometabolic risk factors was examined using receiver operating characteristic (ROC) analyses. The DeLong test and Z test were used for the comparisons of areas under ROC curves (AUCs).

**Results:**

The prevalence of impaired fasting glucose, dyslipidemia, hypertension and cluster of risk factors were 2.9%, 27.3%, 10.5% and 5.7% respectively. The four anthropometric indices showed poor to fair discriminatory ability for cardiometabolic risk factors with the AUCs ranging from 0.53–0.72. Each index performed significantly better AUCs for dyslipidemia (0.59–0.63 vs. 0.56–0.59), hypertension (0.62–0.70 vs. 0.55–0.65) and clustered risk factors (0.70–0.73 vs. 0.60–0.64) in boys than that in girls. BMI percentile performed the best accuracy for hypertension in both sexes; WC percentile had the highest AUC for dyslipidemia and BMI percentile and waist-height ratio performed similarly the best AUCs for clustered risk factors in boys while BMI percentile, WC percentile and waist-height ratio performed similar and better AUCs for dyslipidemia and clustered risk factors in girls; whereas waist-hip ratio was consistently the poorest predictor for them regardless of sex. Though the anthropometric indices were more predictive of dyslipidemia, hypertension and clustered risk factors in overweight/obese group compared to their normal BMI peers, the AUCs in overweight/obese group remained in the poor range below 0.70.

**Conclusions:**

Anthropometric indices are not effective screening tools for pediatric cardiometabolic risk factors, even in overweight/obese children.

## Introduction

Cardiometabolic risk factors among children and adolescents, including hyperglycemia, dyslipidemia, hypertension, etc, have increased with the global pandemic of childhood obesity over recent decades[1]. Cardiometabolic risk factors in childhood are associated with earlier onset and greater risk of many chronic disorders in adults such as cardiovascular disease, metabolic syndrome and type 2 diabetes[2–4]. Thus, early screening of cardiometabolic risks is believed to be crucial for the prevention and intervention of chronic diseases[5].

Although cardiometabolic risk factors are mostly determined by objective approaches (e.g., laboratory tests), non-invasive and easy anthropometric measurements, such as body mass index (BMI) and waist circumference (WC), have been proposed as feasible alternatives for assessing cardiometabolic risks in early stages because of the robust relationship between childhood obesity and cardiometabolic risks[6–8]. However, existing studies have reported controversial results for the predictive capabilities of anthropometric indices for cardiometabolic risk factors among children and adolescents[9–16]. Some studies have suggested that certain, not all, anthropometric indices were useful screening tools for identifying children and adolescents with elevated cardiometabolic risk[9–13]; on the contrary, other studies disapproved of anthropometric indices for predicting pediatric cardiometabolic risks due to the poor accuracy observed[14–16]. In these studies, the discriminatory ability of BMI, WC, and waist-height ratio for cardiometabolic risk factors have been studied a lot while there were few studies on waist-hip ratio, a commonly used index for central obesity in adults. Furthermore, current research mainly focused on general population or overweight/obese children, there is however little evidence on the predictive performance of anthropometric indices for cardiometabolic risk factors among children and adolescents with different BMI categories. Therefore, further research is warranted to investigate the predictive accuracy of anthropometric indices for screening cardiometabolic risks.

This study aimed to comprehensively assess the discriminatory ability of four commonly used anthropometric indices (BMI percentile, WC percentile, waist-height ratio and waist-hip ratio) for identifying cardiometabolic risk factors in Chinese children and adolescents stratified by sex and BMI categories. Findings of this study will contribute to a better understanding of the effectiveness of those indices in predicting cardiometabolic risks and inform future preventive practices by guiding how to choose anthropometric measurements for screening cardiometabolic risks without caution.

## Methods and materials

### Study design and participants

This study was based on a national cross-sectional survey conducted during September and December 2013 in seven provinces in China–Liaoning Province (Northeast), Tianjin Municipality (North), Shanghai Municipality (East), Hunan Province (Central), Guangdong Province (Southeast), Ningxia Autonomous Region (Northwest), and Chongqing Municipality (Southwest). The protocol has been described elsewhere[17]. Briefly, a multi-stage stratified cluster sampling method was used to recruit primary and secondary students: 4–10 primary schools, 2–6 junior high schools, and 2–6 senior high schools were selected in each province; 15–25 classes were randomly chosen from each of Grades 1–12 in the selected schools, except Grades 6, 9, and 12 to avoid influences on their preparation for graduation examination. 65347 students from 94 schools in seven provinces were enrolled in the physical examination. According to the protocol, only two primary school, one junior school and one senior high school were randomly selected from each province because of limited funding, and the students in those selected schools were invited for blood collection. Finally, 16756 students participated in blood examination, including 2160 from Hunan, 2471 from Ningxia, 2770 from Tianjin, 2163 from Chongqing, 2338 from Liaoning, 2316 from Shanghai and 2538 from Guangzhou. Those with missing anthropometric measurements (n = 694), blood pressures (n = 92), fasting plasma glucose (n = 10), or lipid levels (n = 3) and outliers of these measurements (n = 259) were excluded from this study. The outliers were defined as measurements higher than the sum of Q3 plus 3 times interquartile range or measurements lower than the difference of Q1 minus 3 times interquartile range in each sex-age group in boxplots. A total of 15698 children and adolescents were included in following analyses. The study was approved by the Ethical Committee of the Peking University (NO.IRB00001052-13034). Written informed consents were obtained from each student and their parents.

### Anthropometric measurements

Height, weight, waist and hip circumferences of all participants were measured by experienced technicians in accordance with standard procedures. The standing height was measured to the nearest 0.1 cm using a fixed stadiometer (model RGT-140, China), and body weight was measured using a lever-type weight scale to the nearest 0.1 kg (model TZG, China). Waist and hip circumferences were also measured to the nearest 0.1 cm.

### Cardiometabolic measurements

Blood pressures were measured by trained medical staff with mercury sphygmomanometers (model XJ11D, China), stethoscopes (model TZ-1, China), and appropriate cuffs. Participants were asked to sit quietly for at least 5 min prior to the first reading. Systolic blood pressure (SBP) was determined by onset of the first Korotkoff sound and diastolic blood pressure (DBP) was determined by the fifth Korotkoff sound. Blood pressure was measured twice with 5-min gap between two measurements and the mean values were calculated.

After an overnight fast of 12 h, venous blood samples (5ml) were obtained from the antecubital vein of each participant and collected into EDTA vacuum tubes between 7 and 9 AM. Samples were centrifuged at 3000r, aliquoted and stored at -80°C. Levels of fasting plasma glucose (FPG), total cholesterol (TC), low-density lipoprotein cholesterol (LDL), high-density lipoprotein cholesterol (HDL), and triglyceride (TG) were determined at a validated biomedical analyses company, which is accredited by Peking University. The FPG level was measured by glucose oxidase method; TC and TG levels were measured by enzymatic methods; and LDL and HDL levels were measured by clearance method. The non-high-density lipoprotein cholesterol (nHDL) level was calculated by subtracting HDL level from TC level.

### Adiposity-related anthropometric indices

Age- and sex-specific BMI percentiles were calculated based on the BMI growth charts for Chinese children and adolescents[18]. Overweight and obesity were defined based on the age-sex-specific BMI cut-offs equivalent to BMI ≥24 kg/m^2^ and BMI ≥28 kg/m^2^ at 18 years of age, respectively[18].

Age- and sex-specific WC percentiles were calculated based on the WC growth charts for Chinese children and adolescents[19].

The waist-height ratio was calculated as dividing waist circumference by height.

The waist-hip ratio was calculated as dividing waist circumference by hip circumference.

### Definition of cardiometabolic risk factors

Cardiometabolic risk factors were determined based on recommended definitions for children and adolescents identified in the literatures. According to 2011 Expert Panel on Integrated Guidelines for Cardiovascular Health and Risk Reduction in Children and Adolescents[5], abnormal lipid levels were determined as follows: TC ≥5.18 mmol/L; nHDL ≥3.76 mmol/L; LDL ≥3.37 mmol/L; TG ≥1.13 mmol/L for 0–9 years and ≥1.47 mmol/L for 10–19 years; HDL <1.04 mmol/L. Dyslipidemia was defined as the presence of one or more of the five conditions above.

Impaired fasting glucose (IFG) was defined as FPG ≥5.6 mmol/L[20].

High SBP and high DBP was defined as SBP and DBP at or above the 95^th^ percentile based on age and sex respectively, and hypertension was determined as high SBP or high DBP[21].

Cluster of cardiometabolic risk factors was created as accumulation of three or more risk factors above, i.e., high TC, high nHDL, high LDL, high TG, low HDL, IFG, high SBP and high DBP.

### Statistical analyses

The normality of continuous data was examined by Lilliefors and Shapiro-Wilk tests. All continuous variables didn’t conform to normal distribution and were described by median and quartile. The Mann-Whitney U test, *t* test, and chi-square test were used for comparing anthropometric indices and cardiometabolic risk factors between sexes. Partial correlations were performed between cardiometabolic risk factors and anthropometric indices adjusting for age and sex. The interactions between anthropometric indices and sex were analyzed by using logistic regression models with each cardiometabolic risk factor as dependent variables and so were the interactions between anthropometric indices and BMI categories. Receiver operating characteristic (ROC) analyses were used to assess the predictive performance of anthropometric indices for cardiometabolic risk factors. The area under the ROC curve (AUC), which ranges from 0.5 to 1.0, provides a measure of the model’s discriminatory ability. In general, if AUC = 0.5: this suggests no discrimination; if 0.5<AUC<0.7: this is considered poor discrimination; if 0.7≤AUC<0.8: this is considered acceptable discrimination; if 0.8≤AUC<0.9: this is considered excellent discrimination; if AUC≥0.9: this is considered outstanding discrimination[22]. The AUCs of four anthropometric indices were compared with each other by the DeLong test[23] and the comparisons of AUCs between sexes or BMI categories were performed by Z test. We didn’t perform weighted analysis in our study because the aim of this study was to find associations at an individual level and not to report population estimates[24]. ROC analyses and comparisons were conducted in MedCalc (MedCalc Software bvba, Ostend, Belgium), and other statistical analyses were conducted in the SPSS 19 statistical package (SPSS Inc, Chicago, Illinois).

## Results

### Basic characteristics of study participants

The levels of weight, height, waist and hip circumferences had no significant differences between excluded and included participants in most of sex-age groups among 65347 students (S1 Table). The demographic characteristics, anthropometric indices, and cardiometabolic risk factors of the included participants were presented in Table 1. The schoolchildren aged from 6–17 years. The overweight and obese rates were 15.7% and 11.8% respectively, with a larger proportion of boys in overweight/obese group relative to the normal weight group. BMI percentile and WC percentile were significantly higher in girls than that of boys, and waist-height ratio and waist-hip ratio were significantly higher in boys. Girls had higher lipid levels but lower FPG and blood pressures compared with boys. The prevalence of IFG, dyslipidemia, hypertension and cluster of cardiometabolic risk factors were 2.9%, 27.3%, 10.5% and 5.7% respectively, with no significant differences between sexes except for higher IFG in boys.

**Table 1:** Demographic characteristics, anthropometric indices and cardiometabolic risk factors in the studied sample.

	Total (n = 15698)	Boys (n = 8004)	Girls (n = 7694)	*P*^
Demographic variables, mean ± SD or *n* (%)				
Age (years)	11.08±3.29	11.08±3.25	11.09±3.34	0.801
Ethnicity				0.916
Han	14697 (93.6)	7492 (93.6)	7205 (93.6)	
Minorities	1001 (6.4)	512 (6.4)	489 (6.4)	
Region				<0.001
Hunan	2116 (13.5)	1188 (14.8)	928 (12.1)	
Ningxia	2013 (12.8)	983 (12.3)	1030 (13.4)	
Tianjin	2683 (17.1)	1314 (16.4)	1369 (17.8)	
Chongqing	2113 (13.5)	1065 (13.3)	1048 (13.6)	
Liaoning	2257 (14.4)	1157 (14.5)	1100 (14.3)	
Shanghai	2165 (13.8)	1108 (13.8)	1057 (13.7)	
Guangzhou	2351 (15.0)	1189 (14.9)	1162 (15.1)	
Home location				0.104
Urban	9489 (60.4)	4888 (61.1)	4601 (59.8)	
Rural	6209 (39.6)	3116 (38.9)	3093 (40.2)	
BMI categories				<0.001
Normal group	11383 (72.5)	5575 (69.7)	5808 (75.5)	
Overweight group	2465 (15.7)	1288 (16.1)	1177 (15.3)	
Obese group	1850 (11.8)	1141 (14.3)	709 (9.2)	
Anthropometric indices, median (quartile)				
BMI percentile	60.64 (32.64–86.86)	57.14 (29.81–87.29)	63.31 (35.57–86.43)	<0.001
WC percentile	65.54 (40.52–87.08)	61.79 (37.45–86.21)	68.79 (44.04–87.70)	<0.001
waist-height ratio	0.43 (0.41–0.47)	0.43 (0.41–0.48)	0.43 (0.41–0.46)	<0.001
waist-hip ratio	0.84 (0.80–0.88)	0.85 (0.81–0.89)	0.83 (0.79–0.87)	<0.001
Cardiometabolic variables, median (quartile)				
FPG (mmol/L)	4.72 (4.39–5.03)	4.78 (4.43–5.09)	4.66 (4.34–4.96)	<0.001
TC (mmol/L)	3.92 (3.47–4.41)	3.86 (3.41–4.35)	3.99 (3.55–4.45)	<0.001
nHDL (mmol/L)	2.55 (2.16–2.99)	2.49 (2.11–2.93)	2.61 (2.23–3.04)	<0.001
LDL (mmol/L)	2.02 (1.68–2.43)	1.99 (1.63–2.41)	2.07 (1.71–2.46)	<0.001
HDL (mmol/L)	1.34 (1.14–1.56)	1.33 (1.13–1.55)	1.35 (1.15–1.56)	0.001
TG (mmol/L)	0.82 (0.63–1.08)	0.78 (0.60–1.04)	0.86 (0.67–1.13)	<0.001
SBP (mmHg)	102.00 (96.00–110.00)	105.00 (98.00–114.00)	101.00 (94.00–110.00)	<0.001
DBP (mmHg)	65.00 (60.00–71.00)	66.00 (60.00–71.00)	64.00 (60.00–70.00)	<0.001
Cardiometabolic risk factors, *n* (%)				
IFG	460 (2.9)	328 (4.1)	132 (1.7)	<0.001
High TC	863 (5.5)	401 (5.0)	462 (6.0)	0.006
High nHDL	835 (5.3)	401 (5.0)	434 (5.6)	0.078
High LDL	483 (3.1)	227 (2.8)	256 (3.3)	0.075
Low HDL	2285 (14.6)	1262 (15.8)	1023 (13.3)	<0.001
High TG	2020 (12.9)	915 (11.4)	1105 (14.4)	<0.001
High SBP	1141 (7.3)	615 (7.7)	526 (6.8)	0.041
High DBP	1026 (6.5)	540 (6.7)	486 (6.3)	0.276
Dyslipidemia	4284 (27.3)	2147 (26.8)	2137 (27.8)	0.181
Hypertension	1655 (10.5)	879 (11.0)	776 (10.1)	0.068
Cluster of risk factors	889 (5.7)	454 (5.7)	435 (5.7)	0.960

^*P* values for the comparisons of these variables between boys and girls.

### Correlation between anthropometric indices and cardiometabolic variables

As shown in Table 2, all the correlation coefficients between anthropometric indices and cardiometabolic variables were statistically significant in total sample as well as in both sexes (*p* values < 0.05). The four anthropometric indices were negatively correlated with HDL, and positively correlated with the other cardiometabolic variables except the negative correlation between waist-hip ratio and DBP in girls. WC percentile, waist-height ratio and BMI percentile had the highest coefficients for FPG, lipid levels, and blood pressures respectively.

**Table 2:** Age- and sex-adjusted partial correlation coefficients between anthropometric indices and cardiometabolic variables.

Indices	FPG	TC	nHDL	LDL	HDL	TG	SBP	DBP
Total								
BMI percentile	0.093	0.074	0.166	0.119	-0.180	0.216	0.290	0.190
WC percentile	0.112	0.069	0.159	0.113	-0.174	0.207	0.279	0.189
waist-height ratio	0.095	0.132	0.234	0.172	-0.189	0.275	0.248	0.167
waist-hip ratio	0.096	0.114	0.182	0.143	-0.123	0.187	0.119	0.047
Boys								
BMI percentile	0.099	0.111	0.210	0.158	-0.190	0.243	0.329	0.208
WC percentile	0.116	0.079	0.185	0.130	-0.209	0.243	0.338	0.232
waist-height ratio	0.099	0.154	0.270	0.196	-0.219	0.327	0.310	0.221
waist-hip ratio	0.115	0.130	0.220	0.166	-0.169	0.249	0.178	0.108
Girls								
BMI percentile	0.085	0.029	0.117	0.074	-0.179	0.187	0.256	0.173
WC percentile	0.107	0.056	0.129	0.093	-0.142	0.170	0.221	0.144
waist-height ratio	0.086	0.093	0.187	0.138	-0.180	0.217	0.195	0.110
waist-hip ratio	0.077	0.104	0.145	0.121	-0.068	0.126	0.053	-0.021

Note: All the correlation coefficients between anthropometric indices and cardiometabolic variables were statistically significant regardless of sex (*P* values <0.05).

### The discriminatory ability of anthropometric indices for cardiometabolic risk

In the total sample, the AUCs of four anthropometric indices for cardiometabolic risk factors ranged from 0.53 to 0.72. Among them, only AUCs of BMI percentile and WC percentile for elevated SBP were higher than or equal to 0.70 (Table 3).

**Table 3:** Areas under the ROC curve (AUCs) and 95% confidence intervals of the four anthropometric indices for cardiometabolic risk factors in Chinese children and adolescents according to sex.

Indices	IFG		High TC	High nHDL	High LDL	Low HDL	High TG	High SBP	High DBP	Dyslipidemia	Hypertension	Cluster of risk factors
Total												
BMI percentile	**0.57** **(0.54–0.59)**		**0.55** **(0.53–0.57)**	**0.64** **(0.62–0.66)**	**0.60** **(0.57–0.62)**	**0.59** **(0.58–0.61)**	**0.66** **(0.65–0.68)**	**0.72** **(0.70–0.73)**	**0.65** **(0.63–0.67)**	**0.61** **(0.60–0.62)**	**0.68** **(0.67–0.69)**	**0.69** **(0.67–0.71)**
WC percentile	**0.57** **(0.54–0.60)**		**0.55** **(0.52–0.57)**	**0.64** **(0.61–0.66)**	**0.60** **(0.57–0.63)**	**0.61** **(0.59–0.62)**	**0.66** **(0.64–0.67)**	**0.70** **(0.68–0.72)**	**0.63** **(0.61–0.65)**	**0.61** **(0.60–0.62)**	**0.66** **(0.64–0.67)**	**0.68** **(0.66–0.70)**
waist-height ratio	**0.55** **(0.53–0.58)**		**0.58** **(0.56–0.60)**	**0.65** **(0.63–0.67)**	**0.62** **(0.59–0.65)**	**0.58** **(0.57–0.59)**	**0.66** **(0.64–0.67)**	**0.68** **(0.66–0.69)**	**0.61** **(0.59–0.63)**	**0.61** **(0.60–0.62)**	**0.64** **(0.62–0.65)**	**0.68** **(0.66–0.70)**
waist-hip ratio	**0.54** **(0.51–0.56)**		**0.59** **(0.57–0.61)**	**0.63** **(0.61–0.65)**	**0.64** **(0.61–0.66)**	**0.53** **(0.52–0.55)**	**0.61** **(0.60–0.63)**	**0.62** **(0.60–0.64)**	**0.56** **(0.54–0.58)**	**0.58** **(0.56–0.59)**	**0.58** **(0.57–0.60)**	**0.65** **(0.63–0.67)**
Boys												
BMI percentile	**0.56** **(0.53–0.60)** ^**c**^	**0.59** **(0.56–0.63)** ^**#**^^**a**^^**b**^^**c**^		**0.68** **(0.65–0.71)**^**#**^^**a**^^**b**^	**0.65** **(0.61–0.69)**^**#**^^**a**^^**b**^^**c**^	**0.59** **(0.57–0.61)**^**a**^^**c**^	**0.69** **(0.67–0.71)**^**#**^^**c**^	**0.74** **(0.72–0.76)**^**#**^^**b**^^**c**^	**0.67** **(0.64–0.69)**^**#**^^**a**^^**c**^	**0.62** **(0.61–0.64)**^**#**^^**a**^^**c**^	**0.70** **(0.68–0.72)**^**#**^^**a**^^**b**^^**c**^	**0.73** **(0.70–0.76)**^**#**^^**a**^^**c**^
WC percentile	**0.57** **(0.54–0.60)**^**d**^^**e**^	**0.56** **(0.53–0.60)**^**d**^^**e**^		**0.66** **(0.63–0.69)**^**#**^^**d**^	**0.63** **(0.58–0.67)**^**#**^^**d**^^**e**^	**0.62** **(0.60–0.64)**^**#**^^**d**^^**e**^	**0.69** **(0.67–0.71)**^**#**^^**e**^	**0.73** **(0.71–0.76)**^**#**^^**d**^^**e**^	**0.66** **(0.63–0.68)**^**#**^^**e**^	**0.63** **(0.62–0.65)**^**#**^^**d**^^**e**^	**0.69** **(0.67–0.71)**^**#**^^**d**^^**e**^	**0.72** **(0.69–0.75)**^**#**^^**d**^^**e**^
waist-height ratio	**0.55** **(0.51–0.58)**	**0.62** **(0.59–0.65)**^**#**^^**f**^		**0.69** **(0.66–0.72)**^**#**^	**0.67** **(0.63–0.71)**^**#**^^**f**^	**0.58** **(0.56–0.60)**^**f**^	**0.70** **(0.68–0.72)**^**#**^^**f**^	**0.71** **(0.69–0.73)**^**#**^^**f**^	**0.66** **(0.63–0.68)**^**#**^^**f**^	**0.62** **(0.61–0.64)**^**#**^^**f**^	**0.68** **(0.66–0.70)**^**#**^^**f**^	**0.73** **(0.70–0.76)**^**#**^^**f**^
waist-hip ratio	0.53 (0.50–0.56)	**0.64** **(0.61–0.66)**^**#**^		**0.69** **(0.66–0.71)**^**#**^	**0.69** **(0.66–0.73)**^**#**^	**0.53** **(0.52–0.55)**	**0.66** **(0.64–0.68)**^**#**^	**0.65** **(0.62–0.67)**^**#**^	**0.59** **(0.57–0.62)**^**#**^	**0.59** **(0.58–0.61)**^**#**^	**0.62** **(0.59–0.64)**^**#**^	**0.70** **(0.67–0.72)**^**#**^
Girls												
BMI percentile	**0.59** **(0.54–0.64)**^**b**^	0.51 (0.48–0.54)^**b**^^**c**^		**0.60** **(0.57–0.63)**	**0.55** **(0.51–0.59)**^**a**^^**b**^^**c**^	**0.60** **(0.58–0.62)**^**b**^^**c**^	**0.63** **(0.62–0.65)**^**c**^	**0.69** **(0.67–0.71)**^**a**^^**b**^^**c**^	**0.63** **(0.61–0.66)**^**a**^^**b**^^**c**^	**0.59** **(0.57–0.60)**^**c**^	**0.65** **(0.63–0.68)**^**a**^^**b**^^**c**^	**0.64** **(0.61–0.67)**^**c**^
WC percentile	**0.59** **(0.54–0.64)** ^**d**^	0.52 (0.50–0.55)^**d**^^e^		**0.60** **(0.57–0.63)**	**0.57** **(0.53–0.60)**^**e**^	**0.59** **(0.57–0.61)**^**d**^^**e**^	**0.62** **(0.61–0.64)**^**e**^	**0.66** **(0.63–0.68)**^**d**^^**e**^	**0.60** **(0.57–0.63)**^**d**^^**e**^	**0.59** **(0.57–0.60)**^**e**^	**0.62** **(0.60–0.64)**^**d**^^**e**^	**0.64** **(0.61–0.67)**^**e**^
waist-height ratio	0.55 (0.50–0.60)	**0.54** **(0.51–0.57)**^**f**^		**0.61** **(0.59–0.64)**	**0.58** **(0.54–0.61)**^**f**^	**0.58** **(0.56–0.60**^**f**^	**0.63** **(0.61–0.65)**^**f**^	**0.64** **(0.61–0.66)**^**f**^	**0.56** **(0.53–0.59)**^**f**^	**0.59** **(0.57–0.60)**^**f**^	**0.59** **(0.57–0.61)**^**f**^	**0.63** **(0.60–0.66)**^**f**^
waist-hip ratio	0.54 (0.49–0.58)	**0.57** **(0.54–0.60)**		**0.59** **(0.56–0.62)**	**0.61** **(0.58–0.65)**	**0.52** **(0.50–0.54)**	**0.60** **(0.58–0.61)**	**0.59** **(0.56–0.62)**	0.51 (0.49–0.54)	**0.56** **(0.55–0.58)**	**0.55** **(0.52–0.57)**	**0.60** **(0.57–0.63)**

Boldfaced numbers indicate the AUC was statistically greater than 0.50 (*p* < 0.05)

^#^Significant difference for the AUCs between sexes by Z test (*p* < 0.05)

^a^ Significant difference for the AUCs of BMI percentile and WC percentile by Delong test (*p* < 0.05)

^b^ Significant difference for the AUCs of BMI percentile and waist-height ratio by Delong test (*p* < 0.05)

^c^ Significant difference for the AUCs of BMI percentile and waist-hip ratio by Delong test (*p* < 0.05)

^d^ Significant difference for the AUCs of WC percentile and waist-height ratio by Delong test (*p* < 0.05)

^e^ Significant difference for the AUCs of WC percentile and waist-hip ratio by Delong test (*p* < 0.05)

^f^ Significant difference for the AUCs of waist-height ratio and waist-hip ratio by Delong test (*p* < 0.05).

Since the interactions between each anthropometric index and sex were statistically significant for most of cardiometabolic risk factors adjusting for sex and the corresponding anthropometric index (S2 Table), the predictive capabilities of anthropometric indices for cardiometabolic risk were further analyzed by sex.

For IFG, the AUCs of anthropometric indices ranged from 0.53 to 0.57 in boys and 0.54 to 0.59 in girls. The AUC of each index showed no significant differences between sexes. Waist-hip ratio in both sexes and waist-height ratio in girls had no discrimination for IFG. BMI percentile and WC percentile performed similar and better AUCs in both sexes (Table 3).

**Fig 1:**
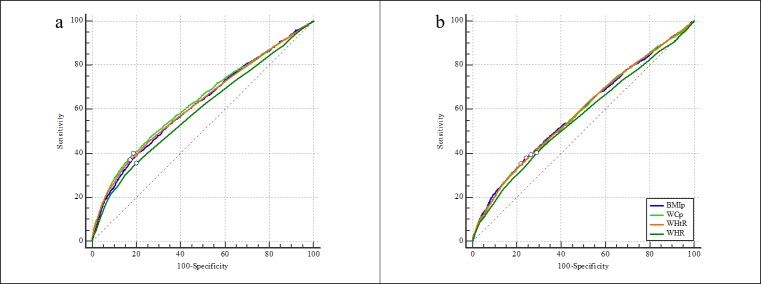
**The AUCs of four anthropometric indices for screening dyslipidemia in boys (a) and girls (b).** The small circle on each ROC curve means the point corresponding to the largest Youden index. BMIp: BMI percentile; WCp: WC percentile; WHtR: waist-height ratio; WHR: waist-hip ratio.

For dyslipidemia, the AUCs of four anthropometric indices ranged from 0.59–0.63 in boys and 0.56–0.59 in girls. Each index performed better AUC for identifying dyslipidemia among boys compared to girls. WC percentile showed the best AUC while waist-hip ratio performed the poorest AUC for dyslipidemia in boys. Similar performance was observed by BMI percentile, WC percentile, and waist-height ratio while waist-hip ratio was still the poorest predictor in girls (Table 3 and Fig 1).

**Fig 2:**
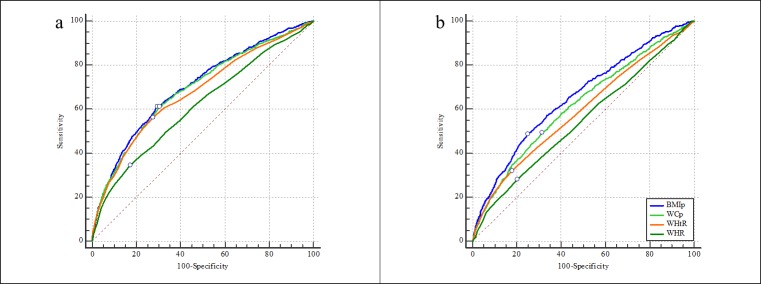
**The AUCs of four anthropometric indices for screening hypertension in boys (a) and girls (b).** The small circle on each ROC curve means the point corresponding to the largest Youden index. BMIp: BMI percentile; WCp: WC percentile; WHtR: waist-height ratio; WHR: waist-hip ratio.

As for hypertension, the AUCs of four anthropometric indices were 0.62–0.70 for boys and 0.55–0.65 for girls. Each index showed better discriminatory ability for hypertension in boys compared to girls. The AUCs of BMI percentile, WC percentile, waist-height ratio, and waist-hip ratio for hypertension were shown in descending order in both sexes (Table 3 and Fig 2).

**Fig 3:**
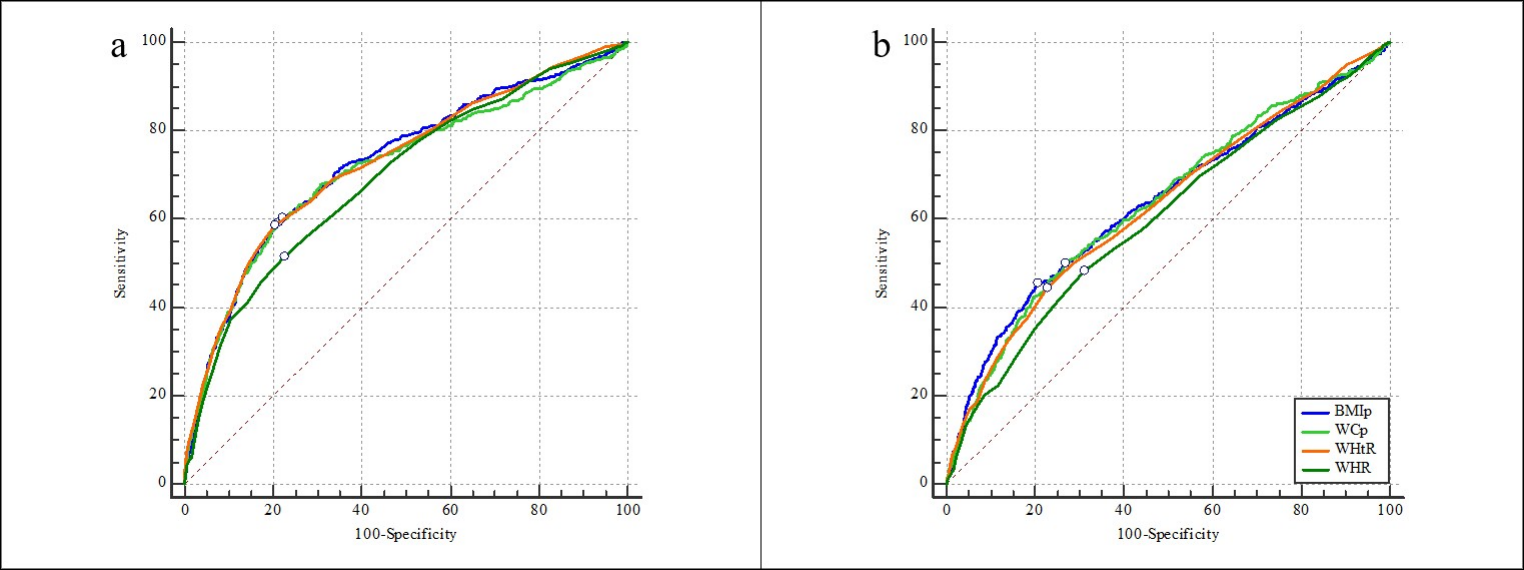
**The AUCs of four anthropometric indices for screening cluster of cardiometabolic risk factors in boys (a) and girls (b).** The small circle on each ROC curve means the point corresponding to the largest Youden index. BMIp: BMI percentile; WCp: WC percentile; WHtR: waist-height ratio; WHR: waist-hip ratio.

With regard to cluster of cardiometabolic risk factors, the four anthropometric indices performed fair discrimination in boys with AUCs from 0.70–0.73 but poor range of AUCs (0.60–0.64) in girls. The performance of each index was significantly better in boys relative to that in girls. BMI percentile and waist-height ratio had similarly the best AUC for cluster of risk factors among boys. Statistically similar AUCs were performed by BMI percentile, WC percentile, and waist-height ratio in girls. Waist-hip ratio was the poorest predictor for cluster of risk factors in both sexes (Table 3 and Fig 3).

### The discriminatory ability of anthropometric indices for cardiometabolic risk by BMI categories

Further analyses were conducted in different BMI categories because the interactions between each anthropometric index and BMI categories were significant for most of risk factors adjusting for the anthropometric index and BMI categories (S3 Table). The anthropometric indices performed quite poor accuracy for cardiometabolic risk factors in normal BMI group with all the AUCs below 0.60. The four anthropometric indices were more predictive of dyslipidemia, hypertension and clustered risk factors in overweight/obese group compared to their normal BMI peers. However, the AUCs in overweight/obese group were also in the poor range below 0.70 and had no advantage for identifying cardiometabolic risk factors compared with that in total sample (S4 Table).

## Discussion

Cardiometabolic risk factors have been an increasing public concern worldwide and also in China. More than a quarter of total sample had abnormal lipids, over one in ten participants were determined as having hypertension and 5.7% of children and adolescents were found to have at least three cardiometabolic abnormalities clustered in our study. Such high prevalence of pediatric cardiometabolic risk factors foreshadows the enormous burden of chronic diseases in Chinese population in the future. Effective screening and intervention of cardiometabolic risk factors in children and adolescents are urgently needed.

To our knowledge, this is the first national study in China which comprehensively assessed the predictive capability of four adiposity-related anthropometric indices (BMI percentile, WC percentile, waist-height ratio and waist-hip ratio) in identifying cardiometabolic risk factors in children and adolescents. By analyzing a large-sample dataset, we found in general the poor accuracy of all four indices in both sexes from the perspective of clinical application.

Our findings were consistent with most of existing studies that anthropometric indices performed poor to fair accuracy for hyperglycemia, dyslipidemia, hypertension and cluster of risk factors. In a recent meta-analysis for AUCs of BMI, WC, and waist-height ratio for pediatric cardiometabolic risk factors, the pooled AUCs for hyperglycemia, elevated TC, elevated TG, low HDL, elevated LDL, hypertension and at least three comorbidities were 0.57–0.57, 0.55–0.56, 0.67–0.73, 0.69–0.70, 0.61–0.62, 0.64–0.68 and 0.69–0.74 respectively[25]. A plausible explanation to the unsatisfactory predictive accuracy is that there are many important factors contributing to levels of fasting glucose, serum lipids, and blood pressures other than adiposity, such as genetic polymorphism and dietary patterns. Secondly, existing research demonstrated that visceral fat content was the primary cause of metabolic disorders[26, 27], and anthropometric indices are just indirect indicators for body weight or fat, whose limited correlation with visceral fat content during childhood may be another possible reason for the poor accuracy[28, 29]. Therefore, the utilization of anthropometric indices for identifying cardiometabolic risk factors in children and adolescents should be considered with great caution.

Despite this, given that those four will continue to be practical indices for screening cardiometabolic risks, it is still worth comparing their performance that may vary by cardiometabolic risk factors. The existing studies about the predictive superiority of different anthropometric indices for cardiometabolic risk have not reach an agreement yet. A large multi-center survey of overweight/obese adolescents in Germany, Austria, and Switzerland revealed that BMI standard score was more closely associated with hypertension, while WC standard score was more closely associated with dyslipidemia[30]. Lo et al. found that waist-height ratio, WC, and BMI performed similarly in screening most cardiometabolic risk factors among children and adolescents[25]. In another study, WC consistently showed better predictive capabilities for cardiovascular risk factors compared with waist-height ratio and BMI among children in Guangzhou[12]. And there were some studies considering waist-height ratio the best screening tool for pediatric cardiometabolic risk factors[10]. In our study, BMI percentile performed the best accuracy for hypertension in both sexes; WC percentile had the best AUC for dyslipidemia, and BMI percentile and waist-height ratio performed similarly the best AUCs for clustered risk factors in boys while BMI percentile, WC percentile and waist-height ratio performed similar and better AUCs for dyslipidemia and clustered risk factors in girls; whereas waist-hip ratio was consistently the poorest predictor for these cardiometabolic risk factors. The heterogeneity on the predictive superiority of anthropometric indices may be attributed to the different definitions of anthropometric indices and outcome variables, and the racial and regional differences in participants. For instance, BMI and WC can be used for analyses in the form of absolute index and relative index such as percentiles or Z scores. Besides, it is likely that some anthropometric indices of fat distribution among adults, such as waist-hip ratio, may be inappropriate for children and adolescents because of the small amount of visceral fat before adulthood and rapid changes in fat patterning during growth and development[31, 32].

Previous studies have shown that the magnitude of associations between anthropometric variables and cardiometabolic risk factors was greater in overweight and obese group compared with their normal weight peers[33, 34]. Similar findings were observed in our study that anthropometric indices were more predictive of cardiometabolic risk factors among overweight/obese children. However, the AUCs in overweight/obese group remained in the poor range below 0.70 and had no significant advantage of the accuracy of anthropometric indices for cardiometabolic risk factors compared with the corresponding AUCs in total sample, in other words, the combination of overweight/obese BMI categories and elevated BMI percentile, WC percentile, waist-height ratio or waist-hip ratio could not produce greater insight into cardiometabolic risk in our study. This is consistent with the findings by Bauer et al[11]. Some other studies also found that anthropometric indices could not identify cardiometabolic risk factors well among overweight/obese children. For example, a study of obese Italian children and adolescents demonstrated that anthropometric indices (BMI, BMI Z-score, WC, and waist-height ratio) were not satisfactory predictors for metabolic comorbidities with the significant AUCs ranging from 0.55–0.70[14]. Since the vast majority of children and adolescents with a normal BMI category had low levels of WC while overweight/obese subjects were more likely to be central obesity[34], although cardiometabolic risk factors were more popular among overweight/obese children, the discriminatory ability of WC percentile, waist-height ratio or waist-hip ratio didn’t increase because of the smaller intervals of these anthropometric variables. In this sense, other effective screening tools should be used in overweight/obese children and adolescents for cardiometabolic risk assessment, maybe regular blood tests as recommended in the guidelines[5, 35].

Several limitations of the present study should be addressed. First, the analyses of impaired fasting glucose just partially reflected the glycometabolic status of children and adolescents, other important metabolic variables were not be considered in our study, such as insulin resistance index. Second, our study was cross-sectional and could not obtain the data about the duration of obesity and the recent change of body weight, which may also affect blood pressure, glucose and lipid metabolism besides the present weight status. More prospective cohort studies are needed to explore the association between anthropometric indices and cardiometabolic risks before definitive conclusions can be made.

Despite the limitations, this is the first study from a national school-based survey to assess the predictive value of BMI percentile, WC percentile, waist-height ratio and waist-hip ratio for cardiometabolic risk factors in Chinese children and adolescents. Our study provided large-sample evidence that adiposity-related anthropometric indices lack of sufficient predictive capability for cardiometabolic risks in children and adolescents, even in overweight/obese group. It implies that anthropometric indices should be used cautiously for early screening of cardiometabolic risk factors in children and adolescents. More effective indicators or models considering multiple determinants of cardiometabolic risk could be explored in future research.

## Supporting information

S1 Table The distribution of anthropometric measurements between the excluded and included participants by sex and age. (DOCX)

S2 Table The P values for the interactions between each anthropometric index and sex for cardiometabolic risk factors in logistic regression models adjusting for sex and the corresponding anthropometric index. (DOCX)

S3 Table The P values for the interactions between each anthropometric index and BMI categories for cardiometabolic risk factors in logistic regression models adjusting for BMI categories and the corresponding anthropometric index. (DOCX)

S4 Table Areas under the ROC curve (AUCs) and 95% confidence intervals of the four anthropometric indices for cardiometabolic risk factors in children and adolescents by BMI categories. (DOCX)

S5 Table Areas under the ROC curve (AUCs) and 95% confidence intervals of the four anthropometric indices for cardiometabolic risk factors among 15957 children and adolescents in the sensitivity analyses that included the outliers. (DOCX)

## Abbreviations

BMI: body mass index
WC: waist circumference
FPG: fasting plasma glucose
SBP: systolic blood pressure
DBP: diastolic blood pressure
TC: total cholesterol
LDL: low-density lipoprotein cholesterol
HDL: high-density lipoprotein cholesterol
TG: triglyceride
nHDL: non-high-density lipoprotein cholesterol
IFG: impaired fasting glucose
ROC: receiver operating characteristic
AUC: area under ROC curve
