# Supplementary material for: The predictive value of anthropometric indices for cardiometabolic risk factors in Chinese children and adolescents: A national multicenter school-based study
Source: PLoS One. 2020 Jan 21;15(1):e0227954. doi: 10.1371/journal.pone.0227954 (PMC6974264; doi:10.1371/journal.pone.0227954)
Supplement: S1 Table — (DOCX) [file pone.0227954.s001.docx]

S1 Table. The distribution of anthropometric measurements between the excluded and included participants by sex and age.

| Sex | Age (years) | Weight (kg) | |  | Height (cm) | |  | Waist circumference (cm) | |  | Hip circumference (cm) | |
| --- | --- | --- | --- | --- | --- | --- | --- | --- | --- | --- | --- | --- |
|  |  | Excluded | Included |  | Excluded | Included |  | Excluded | Included |  | Excluded | Included |
| Boys | 6 | 24.40±5.25 | 24.63±5.21 |  | 121.59±5.31 | 121.39±5.35 |  | 55.27±6.59 | 55.31±6.55 |  | 63.36±6.12 | 63.18±6.01 |
|  | 7 | 27.22±5.95 | 27.33±6.22 |  | 127.02±5.76 | 127.35±5.91 |  | 57.48±7.56 | 57.45±7.65 |  | 66.10±6.71 | 65.82±7.14 |
|  | 8 | 30.94±7.55 | 30.44±7.84 |  | 132.48±6.03 | 132.33±6.34 |  | 60.47±8.78 | 60.17±8.90 |  | 69.35±7.34 | 68.80±7.94 |
|  | 9 | 34.51±8.69 | 34.38±9.01 |  | 137.43±6.43 | 137.52±6.81 |  | 63.11±9.56 | 63.19±9.68 |  | 72.47±7.93 | 72.30±8.03 |
|  | 10 | 38.66±10.01 | 38.45±10.44 |  | 142.66±7.05 | 142.76±7.13 |  | 66.17±10.39 | 66.07±10.42 |  | 75.57±8.42 | 75.21±8.41 |
|  | 11 | 41.59±11.21 | 41.10±11.40 |  | 146.35±7.54 | 146.64±7.41 |  | 67.57±10.96 | 66.53±10.57 |  | 77.59±8.89 | 77.04±8.52 |
|  | 12 | 49.67±13.49^#^ | 47.39±11.75 |  | 156.53±8.61^#^ | 155.56±8.68 |  | 70.86±11.34^#^ | 68.64±10.23 |  | 82.52±9.30^#^ | 81.22±8.48 |
|  | 13 | 53.39±13.63 | 53.95±13.83 |  | 161.84±8.35 | 162.22±8.62 |  | 71.63±10.84 | 71.40±11.04 |  | 84.75±8.94 | 85.03±8.74 |
|  | 14 | 54.90±12.22 | 54.59±11.62 |  | 165.33±7.67 | 164.95±7.35 |  | 71.29±9.75 | 70.94±9.23 |  | 85.91±7.85 | 85.85±7.49 |
|  | 15 | 60.09±12.74 | 61.10±13.50 |  | 170.24±6.45^#^ | 170.90±6.17 |  | 73.08±9.97 | 73.88±10.51 |  | 89.18±8.12 | 89.33±8.07 |
|  | 16 | 62.77±13.01^#^ | 64.12±12.86 |  | 172.00±6.32^#^ | 172.60±5.63 |  | 74.27±10.08^#^ | 75.56±10.42 |  | 90.85±7.66 | 91.26±7.77 |
|  | 17 | 62.84±12.86^#^ | 65.23±13.39 |  | 172.31±6.10 | 172.92±5.94 |  | 74.25±10.40^#^ | 76.24±10.59 |  | 91.01±7.71 | 91.72±7.90 |
| Girls | 6 | 22.66±4.04^#^ | 23.38±5.38 |  | 120.45±5.09 | 120.14±5.43 |  | 53.25±5.47 | 53.68±5.93 |  | 62.06±5.05 | 62.23±5.70 |
|  | 7 | 25.50±5.31 | 25.34±4.99 |  | 125.90±5.54 | 126.19±5.38 |  | 55.44±6.79 | 55.16±6.11 |  | 65.20±6.14^#^ | 64.47±5.94 |
|  | 8 | 28.81±6.23 | 28.68±6.43 |  | 131.70±6.08 | 131.71±6.43 |  | 57.70±7.23 | 57.73±7.39 |  | 68.11±6.64 | 67.61±6.73 |
|  | 9 | 32.28±7.25 | 32.25±7.43 |  | 137.24±6.75 | 137.77±6.86 |  | 60.60±8.33 | 60.04±8.17 |  | 71.35±7.23 | 70.83±7.18 |
|  | 10 | 36.91±8.55 | 37.15±9.37 |  | 143.86±7.38 | 144.17±7.58 |  | 62.84±8.54 | 63.07±8.64 |  | 75.04±7.70 | 74.91±8.02 |
|  | 11 | 41.67±10.11 | 40.70±9.37 |  | 149.61±7.56 | 148.95±7.60 |  | 64.89±8.91 | 65.34±8.44 |  | 78.83±8.20 | 78.42±8.12 |
|  | 12 | 46.69±10.40 | 46.26±9.43 |  | 155.63±6.35 | 155.30±6.49 |  | 67.44±8.63 | 67.18±7.81 |  | 83.51±7.79 | 83.17±7.27 |
|  | 13 | 49.05±9.51 | 49.58±9.62 |  | 157.57±5.69^#^ | 158.30±5.82 |  | 69.01±8.21 | 68.73±8.01 |  | 85.83±6.94 | 85.77±6.81 |
|  | 14 | 50.56±9.33 | 49.81±7.41 |  | 158.30±5.80 | 158.04±5.37 |  | 69.79±7.96 | 69.22±6.64 |  | 87.47±6.69^#^ | 86.08±5.57 |
|  | 15 | 52.28±8.63 | 52.61±8.49 |  | 159.86±5.48 | 159.73±5.49 |  | 70.63±7.46 | 70.91±7.40 |  | 89.17±6.23^#^ | 88.48±6.03 |
|  | 16 | 53.41±9.47^#^ | 54.62±9.32 |  | 160.23±5.53 | 160.52±5.65 |  | 70.91±7.66^#^ | 71.89±8.08 |  | 89.74±6.46 | 89.99±6.63 |
|  | 17 | 53.64±9.57 | 54.78±9.07 |  | 159.99±5.37 | 160.20±5.74 |  | 70.64±7.80 | 71.35±8.27 |  | 89.90±6.47 | 90.07±6.25 |

^#^*P* values <0.05 for comparisons of anthropometric measurements between excluded and included participants by *t* test.
