# Supplementary material for: The predictive value of anthropometric indices for cardiometabolic risk factors in Chinese children and adolescents: A national multicenter school-based study
Source: PLoS One. 2020 Jan 21;15(1):e0227954. doi: 10.1371/journal.pone.0227954 (PMC6974264; doi:10.1371/journal.pone.0227954)
Supplement: S2 Table — (DOCX) [file pone.0227954.s002.docx]

S2 Table. The *P* values for the interactions between each anthropometric index and sex for cardiometabolic risk factors in logistic regression models adjusting for sex and the corresponding anthropometric index.

| Indices | IFG | High TC | High nHDL | High LDL | Low HDL | High TG | High SBP | High DBP | Dyslipidemia | Hypertension | Cluster of risk factors |
| --- | --- | --- | --- | --- | --- | --- | --- | --- | --- | --- | --- |
| Sex*BMIp | 0.403 | <0.001 | <0.001 | <0.001 | 0.199 | 0.001 | 0.023 | 0.175 | 0.005 | 0.016 | <0.001 |
| Sex*WCp | 0.395 | 0.102 | 0.007 | 0.035 | 0.017 | <0.001 | <0.001 | 0.003 | <0.001 | <0.001 | <0.001 |
| Sex*WHtR | 0.222 | 0.002 | 0.032 | 0.018 | 0.135 | 0.021 | 0.029 | 0.001 | 0.076 | <0.001 | 0.001 |
| Sex*WHR | 0.158 | <0.001 | <0.001 | <0.001 | 0.068 | <0.001 | <0.001 | <0.001 | 0.001 | 0.009 | <0.001 |

BMIp: BMI percentile, WCp: WC percentile, WHtR: waist-height ratio, WHR: waist-hip ratio.
