# Supplementary material for: The predictive value of anthropometric indices for cardiometabolic risk factors in Chinese children and adolescents: A national multicenter school-based study
Source: PLoS One. 2020 Jan 21;15(1):e0227954. doi: 10.1371/journal.pone.0227954 (PMC6974264; doi:10.1371/journal.pone.0227954)
Supplement: S3 Table — (DOCX) [file pone.0227954.s003.docx]

S3 Table. The *P* values for the interactions between each anthropometric index and BMI categories for cardiometabolic risk factors in logistic regression models adjusting for BMI categories and the corresponding anthropometric index.

| Indices | IFG | High TC | High nHDL | High LDL | Low HDL | High TG | High SBP | High DBP | Dyslipidemia | Hypertension | Cluster of risk factors |
| --- | --- | --- | --- | --- | --- | --- | --- | --- | --- | --- | --- |
| BMI category*BMIp | 0.256 | <0.001 | <0.001 | <0.001 | <0.001 | <0.001 | <0.001 | <0.001 | <0.001 | <0.001 | <0.001 |
| BMI category*WCp | 0.054 | 0.008 | <0.001 | 0.001 | <0.001 | <0.001 | <0.001 | 0.002 | <0.001 | <0.001 | <0.001 |
| BMI category*WHtR | 0.030 | 0.214 | 0.092 | 0.452 | <0.001 | 0.008 | 0.100 | 0.013 | <0.001 | 0.005 | 0.007 |
| BMI category*WHR | 0.095 | 0.124 | 0.002 | 0.528 | <0.001 | <0.001 | 0.025 | 0.011 | <0.001 | 0.001 | <0.001 |

BMIp: BMI percentile, WCp: WC percentile, WHtR: waist-height ratio, WHR: waist-hip ratio.
