# Supplementary material for: The predictive value of anthropometric indices for cardiometabolic risk factors in Chinese children and adolescents: A national multicenter school-based study
Source: PLoS One. 2020 Jan 21;15(1):e0227954. doi: 10.1371/journal.pone.0227954 (PMC6974264; doi:10.1371/journal.pone.0227954)
Supplement: S4 Table — (DOCX) [file pone.0227954.s004.docx]

S4 Table. Areas under the ROC curve (AUCs) and 95% confidence intervals of the four anthropometric indices for cardiometabolic risk factors in children and adolescents by BMI categories.

| Indices | IFG | High TC | High nHDL | High LDL | Low HDL | High TG | High SBP | High DBP | Dyslipidemia | Hypertension | Cluster of risk factors |
| --- | --- | --- | --- | --- | --- | --- | --- | --- | --- | --- | --- |
| Normal group (n=11383) | | | | | | | | | | | |
| BMI percentile | 0.51  (0.48-0.55) | 0.51  (0.49-0.54) | 0.53  (0.50-0.56) | 0.50  (0.46-0.54) | **0.53**  **(0.51-0.55)** | **0.57**  **(0.56-0.59)** | **0.60**  **(0.58-0.63)** | **0.57**  **(0.54-0.59)** | **0.54**  **(0.52-0.55)** | **0.58**  **(0.56-0.60)** | **0.55**  **(0.52-0.58)** |
| WC percentile | 0.52  (0.48-0.55) | 0.51  (0.49-0.54) | 0.53  (0.50-0.56) | 0.51  (0.47-0.55) | **0.54**  **(0.53-0.56)** | **0.57**  **(0.55-0.58)** | **0.57**  **(0.55-0.60)** | **0.53**  **(0.51-0.56)** | **0.54**  **(0.53-0.56)** | **0.55**  **(0.53-0.57)** | **0.54**  **(0.51-0.57)** |
| waist-height ratio | 0.51  (0.48-0.54) | **0.54**  **(0.51-0.56)** | **0.56**  **(0.53-0.59)** | **0.55**  **(0.52-0.59)** | 0.50  (0.48-0.52) | **0.57**  **(0.55-0.59)** | **0.54**  **(0.51-0.56)** | 0.50  (0.48-0.53) | **0.53**  **(0.52-0.55)** | 0.51  (0.49-0.54) | **0.55**  **(0.52-0.58)** |
| waist-hip ratio | 0.51  (0.48-0.54) | **0.57**  **(0.54-0.59)** | **0.55**  **(0.52-0.58)** | **0.60**  **(0.57-0.64)** | **0.54**  **(0.53-0.56)** | 0.52  (0.50-0.54) | 0.52  (0.49-0.54) | 0.52  (0.50-0.55) | 0.51  (0.49-0.51) | 0.51  (0.49-0.53) | **0.55**  **(0.52-0.58)** |
| Overweight/obese group (n=4315) | | | | | | | | | | | |
| BMI percentile | 0.54  (0.49-0.58) | **0.59**  **(0.55-0.62)^#^** | **0.63**  **(0.60-0.65)^#^** | **0.60**  **(0.57-0.64)^#^** | **0.59**  **(0.57-0.61)^#^** | **0.65**  **(0.63-0.67)^#^** | **0.64**  **(0.61-0.66)** | **0.60**  **(0.57-0.63)** | **0.62**  **(0.61-0.64)^#^** | **0.61**  **(0.59-0.64)^#^** | **0.66**  **(0.64-0.69)^#^** |
| WC percentile | **0.55**  **(0.51-0.59)** | **0.55**  **(0.52-0.58)** | **0.62**  **(0.59-0.64)^#^** | **0.59**  **(0.55-0.62)^#^** | **0.64**  **(0.62-0.66)^#^** | **0.65**  **(0.63-0.67)^#^** | **0.61**  **(0.59-0.63)^#^** | **0.57**  **(0.54-0.60)^#^** | **0.64**  **(0.62-0.65)^#^** | **0.59**  **(0.57-0.61)^#^** | **0.65**  **(0.62-0.67)^#^** |
| waist-height ratio | **0.56**  **(0.51-0.60)** | **0.58**  **(0.55-0.62)^#^** | **0.64**  **(0.61-0.66)^#^** | **0.61**  **(0.58-0.65)^#^** | **0.61**  **(0.59-0.63)^#^** | **0.64**  **(0.62-0.66)^#^** | **0.60**  **(0.57-0.62)^#^** | **0.56**  **(0.54-0.59)^#^** | **0.63**  **(0.61-0.65)^#^** | **0.58**  **(0.56-0.60)^#^** | **0.65**  **(0.63-0.68)^#^** |
| waist-hip ratio | 0.54  (0.50-0.59) | **0.59**  **(0.56-0.63)** | **0.62**  **(0.59-0.65)^#^** | **0.62**  **(0.58-0.66)** | **0.57**  **(0.55-0.59)** | **0.62**  **(0.60-0.64)^#^** | **0.57**  **(0.54-0.59)^#^** | **0.53**  **(0.50-0.56)** | **0.61**  **(0.59-0.62)^#^** | **0.55**  **(0.53-0.57)^#^** | **0.62**  **(0.59-0.65)^#^** |

Boldfaced numbers indicate the AUC was statistically greater than 0.50 (*p* < 0.05). ^#^Significant difference for the AUCs between normal and overweight/obese groups by Z test (*p* < 0.05).
