# Supplementary material for: The predictive value of anthropometric indices for cardiometabolic risk factors in Chinese children and adolescents: A national multicenter school-based study
Source: PLoS One. 2020 Jan 21;15(1):e0227954. doi: 10.1371/journal.pone.0227954 (PMC6974264; doi:10.1371/journal.pone.0227954)
Supplement: S5 Table — (DOCX) [file pone.0227954.s005.docx]

S5 Table. Areas under the ROC curve (AUCs) and 95% confidence intervals of the four anthropometric indices for cardiometabolic risk factors among 15957 children and adolescents in the sensitivity analyses that included the outliers.

| Indices | IFG | High TC | High nHDL | High LDL | Low HDL | High TG | High SBP | High DBP | Dyslipidemia | Hypertension | Cluster of risk factors | |
| --- | --- | --- | --- | --- | --- | --- | --- | --- | --- | --- | --- | --- |
| Total (n=15957) | | | | | | | | | | | |  |
| BMI percentile | **0.57**  **(0.54-0.59)** | **0.55**  **(0.53-0.57)** | **0.64**  **(0.62-0.66)** | **0.57**  **(0.54-0.60)** | **0.59**  **(0.58-0.60)** | **0.66**  **(0.65-0.67)** | **0.72**  **(0.70-0.73)** | **0.65**  **(0.63-0.67)** | **0.60**  **(0.59-0.61)** | **0.68**  **(0.66-0.69)** | **0.69**  **(0.67-0.71)** | |
| WC percentile | **0.57**  **(0.54-0.60)** | **0.55**  **(0.53-0.57)** | **0.64**  **(0.62-0.66)** | **0.57**  **(0.54-0.60)** | **0.60**  **(0.59-0.62)** | **0.66**  **(0.64-0.67)** | **0.70**  **(0.68-0.71)** | **0.63**  **(0.61-0.64)** | **0.61**  **(0.60-0.62)** | **0.66**  **(0.64-0.67)** | **0.68**  **(0.66-0.70)** | |
| waist-height ratio | **0.56**  **(0.53-0.58)** | **0.58**  **(0.56-0.60)** | **0.65**  **(0.63-0.67)** | **0.58**  **(0.55-0.60)** | **0.58**  **(0.57-0.59)** | **0.66**  **(0.64-0.67)** | **0.68**  **(0.66-0.69)** | **0.61**  **(0.59-0.63)** | **0.60**  **(0.59-0.61)** | **0.63**  **(0.62-0.65)** | **0.68**  **(0.66-0.70)** | |
| waist-hip ratio | **0.54**  **(0.51-0.57)** | **0.59**  **(0.58-0.61)** | **0.63**  **(0.61-0.65)** | **0.57**  **(0.54-0.59)** | **0.53**  **(0.52-0.55)** | **0.61**  **(0.60-0.63)** | **0.61**  **(0.60-0.63)** | **0.55**  **(0.53-0.57)** | **0.57**  **(0.56-0.58)** | **0.58**  **(0.56-0.59)** | **0.64**  **(0.62-0.66)** | |
| Boys (n=8127) | | | | | | | | | | | |  |
| BMI percentile | **0.56**  **(0.53-0.60)^c^** | **0.60**  **(0.56-0.63)^#abc^** | **0.68**  **(0.65-0.71)^#ab^** | **0.61**  **(0.58-0.65)^#b^** | **0.59**  **(0.57-0.61)^ac^** | **0.69**  **(0.67-0.71)^#c^** | **0.74**  **(0.72-0.77)^#bc^** | **0.67**  **(0.64-0.69)^#bc^** | **0.62**  **(0.60-0.63)^#ac^** | **0.70**  **(0.68-0.72)^#bc^** | **0.73**  **(0.71-0.76)^#ac^** | |
| WC percentile | **0.57**  **(0.54-0.60)^de^** | **0.56**  **(0.53-0.60)^de^** | **0.67**  **(0.63-0.70)^#de^** | **0.60**  **(0.57-0.64)^#d^** | **0.62**  **(0.60-0.64)^#de^** | **0.69**  **(0.67-0.71)^#de^** | **0.74**  **(0.71-0.76)^#de^** | **0.66**  **(0.63-0.68)^#e^** | **0.63**  **(0.62-0.65)^#de^** | **0.69**  **(0.67-0.71)^#de^** | **0.72**  **(0.69-0.75)^#e^** | |
| waist-height ratio | **0.55**  **(0.52-0.58)** | **0.62**  **(0.59-0.65)^#f^** | **0.69**  **(0.67-0.72)^#^** | **0.63**  **(0.60-0.67)^#^** | **0.58**  **(0.56-0.60)^f^** | **0.70**  **(0.68-0.72)^#f^** | **0.71**  **(0.69-0.73)^#f^** | **0.66**  **(0.63-0.68)^#f^** | **0.62**  **(0.61-0.64)^#f^** | **0.68**  **(0.66-0.70)^#f^** | **0.73**  **(0.70-0.75)^#f^** | |
| waist-hip ratio | 0.53  (0.50-0.56) | **0.64**  **(0.61-0.67)^#^** | **0.69**  **(0.66-0.72)^#^** | **0.63**  **(0.60-0.67)^#^** | **0.53**  **(0.51-0.55)** | **0.66**  **(0.64-0.68)^#^** | **0.64**  **(0.62-0.67)^#^** | **0.59**  **(0.56-0.61)^#^** | **0.59**  **(0.57-0.60)^#^** | **0.61**  **(0.59-0.63)^#^** | **0.69**  **(0.66-0.71)^#^** | |
| Girls (n=7830) | | | | | | | | | | | |  |
| BMI percentile | **0.59**  **(0.54-0.64)^b^** | 0.51  (0.48-0.54)^bc^ | **0.60**  **(0.57-0.63)** | 0.53  (0.49-0.56) | **0.60**  **(0.58-0.62)^bc^** | **0.63**  **(0.62-0.65)^c^** | **0.69**  **(0.66-0.71)^abc^** | **0.63**  **(0.60-0.65)^abc^** | **0.58**  **(0.57-0.60)^c^** | **0.65**  **(0.63-0.67)^abc^** | **0.64**  **(0.61-0.67)^c^** | |
| WC percentile | **0.59**  **(0.54-0.64)^d^** | 0.52  (0.50-0.55)^de^ | **0.60**  **(0.57-0.63)^d^** | 0.53  (0.50-0.57) | **0.59**  **(0.57-0.61)^de^** | **0.62**  **(0.60-0.64)^e^** | **0.65**  **(0.63-0.68)^de^** | **0.59**  **(0.56-0.62)^de^** | **0.58**  **(0.57-0.60)^e^** | **0.61**  **(0.59-0.63)^de^** | **0.63**  **(0.60-0.66)^e^** | |
| waist-height ratio | **0.55**  **(0.50-0.61)** | **0.54**  **(0.52-0.57)^f^** | **0.62**  **(0.59-0.64)** | 0.53  (0.50-0.56) | **0.58**  **(0.56-0.60)^f^** | **0.63**  **(0.61-0.65)^f^** | **0.63**  **(0.61-0.66)^f^** | **0.55**  **(0.52-0.58)^f^** | **0.58**  **(0.57-0.60)^f^** | **0.58**  **(0.56-0.61)^f^** | **0.63**  **(0.60-0.65)^f^** | |
| waist-hip ratio | 0.53  (0.48-0.58) | **0.58**  **(0.55-0.60)** | **0.59**  **(0.57-0.62)** | 0.53  (0.50-0.56) | 0.52  (0.50-0.54) | **0.60**  **(0.58-0.62)** | **0.58**  **(0.56-0.61)** | 0.50  (0.47-0.53) | **0.55**  **(0.54-0.57)** | **0.54**  **(0.52-0.56)** | **0.59**  **(0.56-0.62)** | |

Boldfaced numbers indicate the AUC was statistically greater than 0.50 (*p* < 0.05); ^#^Significant difference for the AUCs between sexes by Z test (*p* < 0.05); ^a^ Significant difference for the AUCs of BMI percentile and WC percentile by Delong test (*p* < 0.05); ^b^ Significant difference for the AUCs of BMI percentile and waist-height ratio by Delong test (*p* < 0.05); ^c^ Significant difference for the AUCs of BMI percentile and waist-hip ratio by Delong test (*p* < 0.05); ^d^ Significant difference for the AUCs of WC percentile and waist-height ratio by Delong test (*p* < 0.05); ^e^ Significant difference for the AUCs of WC percentile and waist-hip ratio by Delong test (*p* < 0.05); ^f^ Significant difference for the AUCs of waist-height ratio and waist-hip ratio by Delong test (*p* < 0.05).
